# Changes in Phenolic Compounds and Antioxidant Capacity of *Artocarpus heterophyllus* Lam. (Jackfruit) Pulp during In Vitro Gastrointestinal Digestion

**DOI:** 10.3390/antiox13010037

**Published:** 2023-12-23

**Authors:** Ming Cheng, Jiali He, Yu Gu, Gang Wu, Lehe Tan, Chuan Li, Fei Xu, Kexue Zhu

**Affiliations:** 1 Spice and Beverage Research Institute, Chinese Academy of Tropical Agricultural Sciences, Wanning 571533, China; 2 School of Food Science and Engineering, Hainan University, Haikou 570228, China; 3 College of Tropical Crop Science, Yunnan Agricultural University, Pu’er 665099, China; 4 Key Laboratory of Processing Suitability and Quality Control of the Special Tropical Crops of Hainan Province, Wanning 571533, China; 5 National Center of Important Tropical Crops Engineering and Technology Research, Wanning 571533, China; 6 Key Laboratory of Nutritional Quality and Health Benefits of Tropical Agricultural Products of Haikou City, Haikou 571100, China

**Keywords:** *Artocarpus heterophyllus* Lam., phenolic compounds, in vitro digestion, antioxidant capacity

## Abstract

An in vitro gastrointestinal digestion model was applied to investigate the effect of digestion on the phenolic compounds and antioxidant capacity of *Artocarpus heterophyllus* Lam. (jackfruit) pulp. The total phenol content (TPC) was determined using Folin–Ciocalteu method, and the antioxidant activities were evaluated by DPPH and ABTS assays. Phenolic compounds were analyzed using ultra-performance liquid chromatography coupled with electrospray ionization, followed by quadrupole time-of-flight mass spectrometry (UPLC-ESI-Q-TOF-MS/MS). The results showed that TPC was significantly higher after gastric digestion. Thirty phenolic compounds (hydroxybenzoic acids and derivatives, hydroxycinnamic acids and derivatives, and flavonoids) were identified. The antioxidant activities of the digested samples varied with the TPC, and there was a correlation between antioxidant activity and TPC. The present study implies that gastrointestinal digestion may improve TPC and increase the amount of free phenolic compounds, mainly related to changes in pH value and digestive enzymes.

## 1. Introduction

*Artocarpus heterophyllus* Lam. (Jackfruit) is a species of the Moraceae (Mulberry) family and is renowned as the “queen of tropical fruits” [1]. The jackfruit plant can reach a height of 20 m, and the fruits are large with different shapes. The fruit weights normally range from 5 to 20 kg, and the largest fruit can weigh up to 50 kg. Jackfruit contains many bioactive compounds, such as dietary fiber, volatile sterols, pectin, carotene, etc. [2]. Nowadays, the polyphenols of jackfruit have received extensive attention [3]. The polyphenols of jackfruit peel include organic acids, phenolic acids, and flavonoids [4].

Fruits and vegetables are the primary sources of dietary phenolics. Phenolic compounds are important secondary metabolites widely used as natural antioxidants, as well as producers of sensory properties such as color and flavor. Moreover, phenolic compounds have many benefits for the human body, such as inhibiting reactive oxygen and nitrogen species, transferring electrons to free radicals, activating antioxidant enzymes, and alleviating oxidative stress and inflammation. Studies have shown that phenolic compounds positively impact various diseases, including diabetes, obesity, cancer, cardiovascular disease, osteoporosis, and neurodegenerative diseases [5,6,7,8,9]. Thus, phenolic compounds may not only have great application potential in the field of food but also in medicine and healthcare.

Due to the drawbacks, including the time-consuming and high cost of animal and human studies, in vitro digestion models are developed to assess the bioaccessibility or absorption of phytochemicals during digestion [10]. Current studies on in vitro simulated digestion have focused on phenolic compounds in food matrices and extracts, such as coffee (*Coffea arabica* L.) pulp, oranges (*Citrus sinensis*), raspberry, and flours from persimmon fruit (*Diospyros kaki*) co-products, among others [11,12,13,14]. Konsue et al. [15] determined the bioaccessibility of phytochemical compounds in jackfruits using simulated in vitro gastrointestinal digestion. Zhu et al. [16] investigated the effects of in vitro saliva, gastric and intestinal digestion on the chemical properties and antioxidant activity of polysaccharides from *Artocarpus heterophyllus* Lam. (Jackfruit) pulp. After in vitro digestion, the digested jackfruit flake has enhanced protection against acrylamide-induced oxidative damage [17]. The antioxidant activities of papaya, jackfruit, and araticum extracts were evaluated using in vitro gastrointestinal digestion [18]. However, the qualitative analyses and molecular structure of phenolic compounds after in vitro gastrointestinal digestion need further investigation.

**Figure 1:**
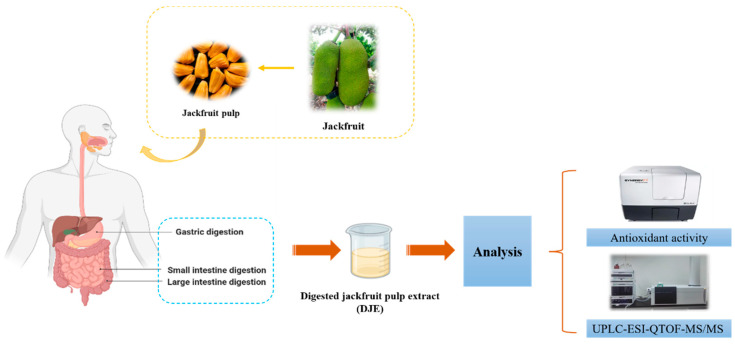
Schematic and design roadmap of in vitro simulated gastrointestinal digestion on phenolic components and the antioxidant activity of jackfruit pulp.

Thus, the present study aimed to investigate the influences of in vitro gastrointestinal digestion on TPC values and antioxidant activity of jackfruit pulp. Phenolic compounds were identified using ultra-performance liquid chromatography coupled with electrospray ionization, followed by quadrupole time-of-flight mass spectrometry (UPLC-ESI-Q-TOF-MS/MS) (Figure 1). The findings may provide new insights into the consumptions of jackfruit, leading to potential health benefits.

## 2. Materials and Methods

### 2.1. Materials and Reagents

Malaysia 1 jackfruit was obtained from the Spice and Beverage Research Institute, Chinese Academy of Tropical Agricultural Sciences (Wanning, Hainan, China). The fruit at the fully ripe stage (14−16 weeks) was selected, and the pulp samples were collected, frozen, and stored at −20 °C for subsequent studies.

Pepsin (from porcine gastric mucosa, ≥500 U/mg), pancreatin (from porcine pancreas), bile salts (porcine bile extract), DPPH (1-diphenyl-2-picrylhydrazyl), and ABTS (2,2′-azinobis (3-ethylbenzothiazoline-6-sulfonic acid) diammonium salt) were from Sigma-Aldrich, Co., Ltd. (St. Louis, MO, USA). Gallic acid was from Shanghai Yuanye Bio-Technology Co., Ltd. (Shanghai, China). Folin-Ciocalteu reagent was from Beijing Solarbio Science & Technology Co.,Ltd. (Beijing, China). All solvents and chemicals utilized were of LC-MS quality or analytical grade (>98%).

### 2.2. In Vitro Gastrointestinal Digestion

Human upper gastrointestinal (GI) digestion was simulated using a two-step in vitro digestion model adapted from the protocol released by INFOGEST [19]. The simulated gastric fluid (SGF) and simulated intestinal fluid (SIF) adhered to the guidelines in the INFOGEST publication (Table S1). To simulate the gastric phase, pepsin solution (0.5 mL, 80,000 U/mL), SGF (8 mL), and CaCl_2_ solution (5 μL, 0.3 mol/L) were mixed in ultra-pure water, and the pH value was adjusted to 3.0 using 1 mol/L HCl. The homogenized jackfruit pulp samples were blended with an identical volume of simulated gastric juice and incubated at 37 °C for 0 h, 1 h, and 2 h, respectively. The digested samples (2.0 mL) were collected and placed in a boiling water bath for 10 min to neutralize them for subsequent assay.

Simulated intestinal digestion was initiated by adding the gastric chyme with SIF (8.5 mL), pancreatin solution (5.0 mL, 100 U/mL), fresh bile solution (2.5 mL, 160 mmol/L), CaCl_2_ (0.04 mL, 0.3 mol/L), and ultrapure water. The pH value was adjusted to 7.0 using 1 mol/L NaOH solution. The mixture solutions were placed in a water bath at 37 °C, and the digested samples (2.0 mL) were collected at 1 h, 2 h, and 4 h, respectively, and then immediately immersed in a boiling water bath for 10 min to stop the digestion.

### 2.3. Extraction of Digested Jackfruit Pulp Samples

Digested jackfruit pulp samples were extracted using an Ultrasonic-microwave Cooperative Extractor/Reactor (Model CW-2000, XTrust Analytical Instrument Technology Co., Ltd., Shanghai, China) according to our previous method [20]. Briefly, digested jackfruit pulp samples and 60% ethanol were mixed and vortexed, at a solid-to-liquid ratio of 1:30 (g: mL). The mixture was then subjected to microwave extraction for 165 s at a power level of 550 W. The extracted blend was centrifugated at 10,000 rpm, 4 °C for 10 min, and the residue was re-extracted twice. The combined supernatants were evaporated using a rotary evaporator R-215 (BUCHI Labortechnik AG, Flawil, Switzerland) to remove ethanol. Subsequently, the concentrate was recovered with ultrapure water until a final volume of 25 mL, and a working solution of digested jackfruit pulp extract (DJE) was obtained.

### 2.4. Determination of Total Phenol Content (TPC)

The Folin–Ciocalteu method was used to measure the phenolic contents. Briefly, DJE (0.5 mL) were mixed with Folin–Ciocalteu reagents (2 mL) for 5 min, and then 7.5% Na_2_CO_3_ (2 mL) was added. A blank was setup by following the same procedure wherein the DJE was replaced with an equal volume of methanol. The absorbance was read at 760 nm after incubation for 40 min in the dark for color development. The results were estimated as gallic acid equivalent (mg GAE/g).

### 2.5. Analysis of Phenolic Compounds

According to our previous method [20], DJE samples were analyzed by Agilent 1290 UPLC and 6530B hybrid Q-TOF-MS (Agilent Technologies, Santa Clara, CA, USA). Mass spectral signals were acquired in positive and negative electrospray ionization (ESI) scanning mode, respectively (Table 1).

### 2.6. Antioxidant Activity Assay

DPPH radical scavenging ability assay. The DPPH method was applied in accordance with a prior report with slight modifications [21]. Briefly, DPPH was dissolved in 80% methanol. A mixture of DPPH working solution (150 μL) and the diluted sample extract (150 μL) or 80% methanol (control) was prepared, shaken, and allowed to stand in the dark at room temperature for 30 min. The absorbance was then read at 517 nm (SynergyH1, BioTek, Santa Clara, CA, USA). Trolox was used as a standard, and the results were presented as μg Trolox equivalent /100 g fresh mass (the calibration curve was Y = −0.0058X + 0.3554, R^2^ = 0.9909).

**ABTS radical scavenging ability assay.** The method of Cheng et al. (2020) was used to determine the ABTS radical scavenging ability [22]. In summary, ABTS solution (7 mmol/L) was combined with potassium persulfate solution (2.45 mmol/L) in equal volumes and reacted in the dark for 12–16 h to produce ABTS+ solution. Prior to usage, the stock solution was appropriately diluted with ultrapure water to obtain an absorbance of 0.70 ± 0.02 at 734 nm. For the assay, the diluted sample (10 μL) was mixed with ABTS+ solution (190 μL) and incubated in the dark for 6 min. Subsequently, the absorbance of the resulting mixture was read at 734 nm. Trolox was used as a standard, and the results were presented as μg Trolox equivalent /100 g fresh mass (the calibration curve was Y = −1.65X + 0.3682, R^2^ = 0.9957).

### 2.7. Statistical Analysis

Experiments were performed in triplicate, and the results were expressed as mean ± standard error of the mean (SEM). Data were analyzed through one-way analysis of variance (ANOVA) in conjunction with Duncan’s multiple range test using SPSS software (version 26.0, SPSS, Inc., Chicago, IL, USA). The *p*-value was set to 0.05 for a significant difference.

The MS spectra were analyzed and converted to compound Exchange Format (.CEF) files with the help of the “find compounds by molecular feature” tool using Agilent Mass Hunter Qualitative Analysis Software (version B.07.00). The exported files were then imported into Mass Profiler Professional (MPP) software (version 14.0, Agilent Technologies, Santa Clara, CA, USA) for further statistical analysis. Alignment parameters were: RT window = 0.5% + 0.1 min, mass window = 10 ppm + 1 mDa. A principal component analysis (PCA) was performed with MPP to visualize the sample groupings at the different stages of in vitro simulated digestion. The resulting entity list was then processed in the ID browser, which allowed for chemical formulas to be generated and searched against a proprietary database.

## 3. Results and Discussion

### 3.1. TPC

**Figure 2:**
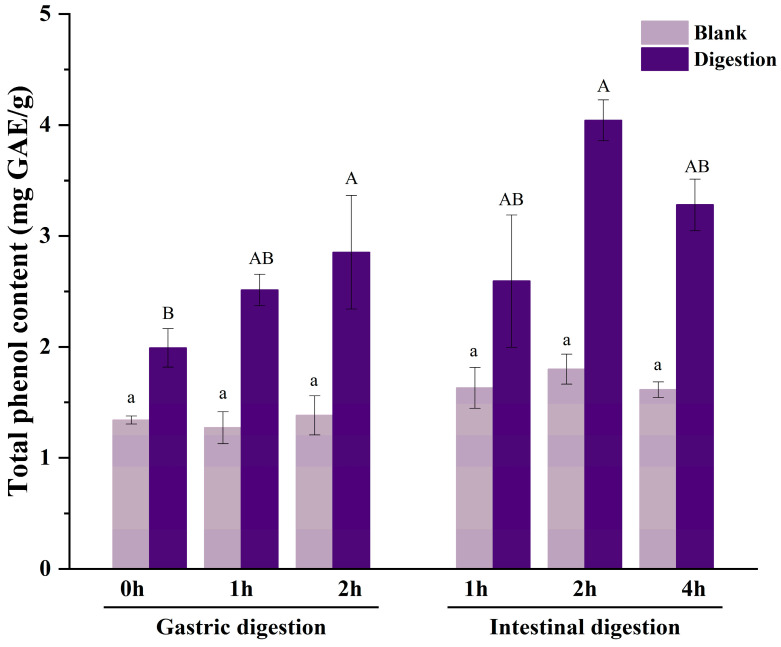
TPC of DJE after in vitro simulated digestion. Different letters point to significant differences in the same digestion solution at different times (*p* < 0.05).

As shown in Figure 2, compared with 0 h of gastric digestion, TPC in jackfruit pulp increased significantly (from 1.99 ± 0.07 to 2.85 ± 0.51 mg GAE/g), with an increase of 43.22% after 2 h of gastric digestion. This result is similar to previous studies, showing that the TPC values exhibited a continuous increase during simulated digestion [15,17]. Konsue et al. [15] reported that TPC values of jackfruit during simulated digestion varied from 4.48 to 124.84 mg GAE/100 g at four ripening stages. TPC also exhibited a significant increase in undigested and digested extracts, measuring 23.3 ± 0.004 and 33.9 ± 0.002 mg GAE/100 g freeze-dried fruit, respectively [18]. Bouayed et al. [23] reported that TPC in fresh apple fruit increased after gastric digestion. Numerous physical factors (temperature, pH value, ion force) and biological factors (bile salts and enzymes) affect phenolic compound stability during in vitro simulated digestion [24,25]. Pepsin can hydrolyze chemical bonds (covalent bonds, hydrogen bonds, etc.) that are formed by polyphenols bound to some macromolecules (such as proteins and carbohydrates) inside and outside the cell, making the polyphenols that were initially bound to these macromolecules free. It also weakens some of the ester bonds where phenolic acids interact with the cell wall, releasing phenolic acids [26]. Meanwhile, in an acidic environment, polyphenols may also undergo hydrolysis, and some glycosides may be converted into aglycones, increasing the phenolic content [27].

However, TPC was significantly reduced (by 9.11%, *p* < 0.05) after 1 h of intestinal digestion. This result is consistent with previous studies on red- and yellow-colored pea shells [28], and sweet orange (*Citrus sinensis*) [29]. This is due to the dilution of polyphenols caused by the addition of intestinal digestive juices, in addition to an increase in pH value and changes in the acid-base environment, resulting in a decrease in polyphenol content [30]. After 2 h of intestinal digestion, TPC significantly increased. This may be related to the action of intestinal digestive enzymes, which decompose bound phenols in the substrate and release free phenols. Then, TPC started to decrease, which could be caused by decreased pancreatic enzyme activity, slow decomposition of bound phenols, or the conversion of free phenols into other compounds [31]. During gastrointestinal digestion, the TPC of the intestinal digest was higher than that of the gastric digest, indicating that gastrointestinal digestion enhanced the release of phenols in DJE.

### 3.2. Identification of Polyphenols and their Decomposition Products after In Vitro Digestion

**Figure 3:**
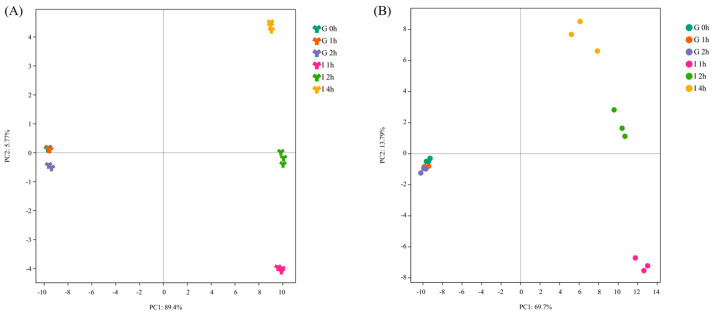
PCA score plot of the metabolites of DJE. (**A**) Positive mode and (**B**) negative mode. G0: Gastric digestion 0 h, G1: Gastric digestion 1 h, G2: Gastric digestion 2 h, I1: Intestinal digestion 1 h, I2: Intestinal digestion 2 h, I4: Intestinal digestion 4 h.

In the PCA analysis (Figure 3), the first two principal components (PC1 and PC2) explained 95.17% of the total variance in the positive mode, while accounting for 83.49% of the total variance in the negative ion mode. The 2D scores plot indicated that the digested jackfruit pulp samples at the different gastric and intestinal digestion stages were clearly separated due to differences in the accumulation of metabolites.

Preliminary identification was achieved by comparing the collected MS data with chemicals previously identified in the literature or registered in Massbank. A total of 30 substances were identified in DJE, including 22 flavonoids (8 flavonols, 5 flavones, and 4 flavanols), 6 hydroxycinnamic acids and derivatives, and 2 hydroxybenzoic acids and derivatives (Table 2). The total ion chromatogram (TIC) of MS spectral data is shown in Figure S1. The MS spectra and structural formulae of the monomeric substances are illustrated in Figure S2.

Hydroxybenzoic acids and derivatives. According to reference substances, compounds 1 and 2 at *m*/*z* 171.0291 [M + H]^+^ and 257.0657 [M + CH_3_COO]^−^, with their resultant ions at *m*/*z* 125 and 153 attributed to the loss of CO_2_, respectively, were identified as gallic and syringic acids, respectively [32].

Hydroxycinnamic acids and derivatives. Compound 3 (Rt = 7.522, *m*/*z* 337.0906) was determined as 5-p-coumaroylquinic acid with a main fragment at *m*/*z* 147 and 119, resulting from the loss of [C_9_H_8_O_2_-H]^−^ and [C_8_H_8_O-H]^−^, respectively. Its fragmentation patterns aligned with previous reports [33,34]. Polyphenols often have multiple *cis*/*trans* isomers. Exposure to UV light leads to phytochemical isomerization of naturally occurring phenols that often appear in the *trans* conformation. O-caffeoylquinic is the main quinate derivative found in jackfruit polyphenols and is often esterified at positions 1, 3, 4, and 5 of quinic acid, yielding four positional isomers. In the negative mode, the MS/MS spectra of caffeoylquinic acid (CQA) typically display common product ions of *m*/*z* 191.06 (C_7_H_12_O_6_), 173.05 (C_7_H_10_O_5_), 179.0342 (C_9_H_8_O_4_), and 135.0049 (C_8_H_8_O_2_). These were attributed to the fragments of [quinic acid-H]^−^, [quinic acid-H_2_O-H]^−^, [caffeic acid-H]^−^, and [caffeic acid-CO_2_-H]^−^, respectively [35,36]. Comparing the retention times and product ion fragments with references from previous studies [37,38], compound 4 exhibited product ions at *m*/*z* 173 and 179, generated by the loss of [quinic acid-H_2_O-H]^−^ and [caffeic acid-H]^−^, and identified as 1-O-caffeoylquinic acid. The C_9_H_7_O_3_ and C_16_H_15_O_7_ in compound 5 were cleaved to produce signals at *m*/*z* 163 and 319; the compound was assigned as (E,E)-3,5-di-O-caffeoylquinic acid based on a comparison with a previous report [39]. In the negative ionization mode, compound 6 (Rt = 1.674 min) had an [M-H]^−^ of *m*/*z* 367.1056 and was proposed to be 3-O-caffeoylquinic acid methyl ester as it lost a hydrogen ion fragment with mass 1 under the negative mode conditions of mass spectrometry. Additionally, the product ion fragments at *m*/*z* 135 could be an adduct ion fragment of C_8_H_7_O_2_ and a hydrogen ion, further supporting the hypothesis that compound 6 may be 3-O-caffeoylquinic acid methyl ester [37]. Similarly, based on the analysis of the fragmentation pattern and database search, compounds 7 and 8 were proposed as 4,5-di-O-caffeoylquinic acid ester and ethyl-3,4-dicaffeoylquinate, respectively.

Flavanols. Flavanols exist in plants as monomers (catechins, epicatechin, epigallocatechin, gallocatechin) or multimers (procyanidins or condensed tannins) [40]. Compound 9 (Rt = 1.563) showed a molecular ion ([M + HCOO]^−^) at *m*/*z* of 353.0869. Based on the mass spectral information and comparison with a study [41], this fragment was considered as the adduct fragment of (+)-catechin and HCOO- ions. This compound generated a diagnostic ion at *m*/*z* 245 [M-CH_2_-CHOH-H]^−^, and so could be (+)-catechin hydrate. Compound 10 demonstrated the [M−H]^−^ ion of *m*/*z* 441.0798 and fragments of *m*/*z* 167, 137, and 125, and so was regarded as (-)-epicatechin gallate. Compound 11 yielded a parent ion at *m*/*z* 459.0919, and the primary fragment ion at *m*/*z* 125 corresponded to a trihydroxybenzene moiety. It was identified as gallocatechin by comparison with a report by Liu et al. (2020) [42]. Compound 12 yielded fragment ions of *m*/*z* 539, 407, and 285, respectively. *m*/*z* 539 is produced by the molecular ion shedding two molecules of H_2_O (36 u). *m*/*z* 407 is produced by the molecular ion undergoing RDA cleavage (152 u) while shedding one more molecule of H_2_O. *m*/*z* 285 corresponds to the molecular ion undergoing quinone methide fission (QM) cleavage (290 u). The molecular ion breakage and fragmentation are consistent with the A-type procyanidin dimer cleavage pattern [43,44]. By mass spectrometry database analysis, compound 12 was identified as procyanidin A1.

Flavonols. Flavonols are the most common flavonoid in food and are represented by kaempferol and quercetin [40]. Compounds 13 and 14 were found to be quercetin derivatives; compound 13 (Rt = 1.58 min, *m*/*z* 397.0781) corresponded to quercetin dihydrate. Meanwhile, precursor ions [M-H]^−^ at *m*/*z* 789.2055 were detected in compound 14, which showed a major fragment ion ([M-H-glycoside]^−^) at *m*/*z* 301. The compound was further identified by database search as quercetin-3-O-beta-D-glucose-7-O-beta-D-gentiobiosiden. Two kaempferol-diglycosides, namely, kaempferol-3-glucoside (compound 15, *m*/*z* 447) and kaempferol 3-O-robinobioside (compound 16, *m*/*z* 595.1632), were identified. Both compounds resulted in the dominant fragment ion at *m*/*z* 285, which was associated with the cleavage of a glycosidic linkage (glucoside or robinobioside) accompanied by a conformational change in H. In addition, compound 17 presented a precursor ion at 625.1758, fragment ions at 314 (C_28_H_32_O_16_); it was then tentatively identified as isorhamnetin-3-O-neohesperidoside. The molecular formula of compound 18 was determined as C_21_H_18_O_14_, by observing the secondary mass spectrometry ion fragmentation. The main fragment ion of the compound was *m*/*z* 493, and by comparison with previous study [45], the compound was identified as hibifolin. Analogously, compounds 19 (C_25_H_26_O_7_) and 20 (C_33_H_40_O_15_) were proposed as papyriflavonol A and sagittatoside A.

Flavanones. Flavanones are generally present as glycosides, and their aglycone form is released during digestion, mainly including hesperidin and naringenin. On the basis of the characteristic fragment ions at *m*/*z* 494 [M + H-C_6_H_10_O_5_], 465 [M + H-C_6_H_10_O_4_], 431 [M + H-C_6_H_10_O_5_-H_2_O], and 303 [M + H-C_6_H_10_O_4_-C_6_H_10_O_5_], compound 21 was proposed as hesperidin, which was in accordance with a relevant report [46]. Compound 22 was assigned as naringenin based on its forming deprotonated molecule at *m*/*z* 271.064 and product ions at *m*/*z* 177 [M-H-C_6_H_5_OH]^−^, 151 (RDA fragmentation reaction cleaved at the C-ring of flavonoid aglycones) and 107 [151-CO_2_]^−^. Similarly, compounds 23 (C_18_H_18_O_5_) and 24 (C_16_H_14_O_5_) were easily assigned to naringenin trimethyl ether and 5-O-Methylnaringenin. The precursor ion [C_25_H_26_O_6_ + HCOO]^−^ at *m*/*z* 467.1715 and fragments at *m*/*z* 367 [C_21_H_20_O_6_-H]^−^, 45 [C_2_H_6_O-H]^−^, and 123 [C_7_H_8_O_2_-H]^−^ were used to identify compound 25 as kuwanol C.

Flavones. Compound 26, with the master ion [M + H]^+^ at *m*/*z* 615.1728, infers that the fragment ion *m*/*z* 593 gives a sodium ion fragment in the positive mode, which is considered to be fortunellin since the fragmentation pattern is comparable to that reported previously [47]. Compound 27 presented a precursor ion at *m*/*z* 395.1096, easily identified as tangeretin. The molecular ion peak [M-H]^−^ of compound 28 was at *m*/*z* 553.1154. In the high mass region, it loses one molecule of CH_3_OH to form the fragment ion with the highest ionic strength *m*/*z* 521, and this fragment ion loses one molecule of CO_2_ to acquire the fragment ion *m*/*z* 477. Therefore, the compound could be inferred to be bilobetin. Based on the analysis of its secondary mass spectrometry ion fragments 271, 243, and 153, compound 29 with [M + H]^+^ at *m*/*z* 289.0703was identified as aromadendrin. Compound 30 (Rt = 2.469, [M + H]^+^ at *m*/*z* 463.0875) confirmed that the fragment ion at *m*/*z* 303 was a 6-hydroxyaluminoenyl protein with a neutral loss of 162, corresponding to the disappearance of the hexose moiety. Hence, it was identified as 6-hydroxytyrosine-7-glucoside.

### 3.3. Antioxidant Activity

**Figure 4:**
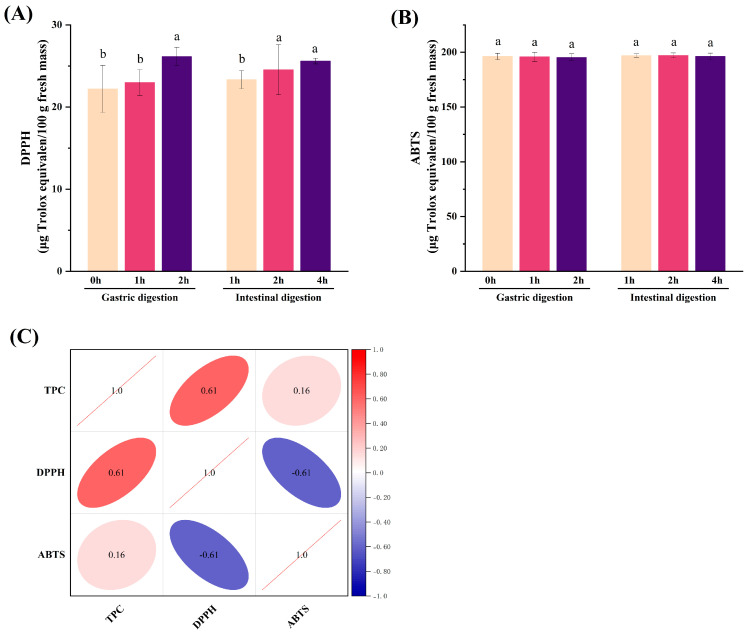
Antioxidant activity (**A**) DPPH radical scavenging ability, (**B**) ABTS radical scavenging ability and the correlation between total phenolic content and antioxidant activity (**C**) of DJE. Different letters indicate significant differences (*p* < 0.05). Positive correlation is indicated by red colors and negative correlation by blue colors.

As shown in Figure 4A, in comparison with the initial gastric digestion stage (22.212 μg Trolox/mL), the DPPH radical scavenging ability of DJE increased by 3.54% and 17.73% after gastric digestion for 1 h and 2 h, respectively, which may be related to the acidic environment conducive to the release of antioxidants. DPPH radical scavenging ability was significantly reduced at 1 h during the intestinal digestion phase (*p* < 0.05), with a 12.07% decrease compared to after gastric digestion. It then started to rise and remained stable at 4 h of intestinal digestion. Pavan et al. [18] reported an increased antioxidant activity of jackfruit extracts after in vitro digestion using trolox equivalent antioxidant capacity and oxygen radical absorbance capacity methods. This may be because of the change in pH value from gastric digestion to intestinal digestion, leading to changes in polyphenolic compounds that affect the free radical scavenging ability of digested extracts [48]. Phenolic compounds are considered critical bioactive compounds in the fight against free radicals. However, no significant differences in ABTS radical scavenging ability were found, needing further investigation.

TPC is closely related to the antioxidant capacity of plants, and bioactive compounds can release monomers or glycosides during gastrointestinal digestion, thereby increasing the number of phenolic hydroxyl groups. This could be due to the interaction between phenolic hydroxyl groups as hydrogen donors and free radicals, boosting their free radical scavenging properties [31,49]. The results of correlation analysis (Figure 4C) showed that the TPC in this study correlated well with the antioxidant capability obtained by the DPPH assay, but not with the antioxidant capacity indicated by the ABTS assay. This is not entirely consistent with a report by Pods Dek et al. (2014) [50]. From this point of view, the link between TPC and antioxidant activity, except for the method of determining antioxidant activity [51], may also be related to the source of phenolic substances, main phenolic components, free or bound state, etc.

## 4. Conclusions

This study focused on the impacts of simulated in vitro gastrointestinal digestion on the TPC, phenolic constituents, and antioxidant activity of DJE. The TPC in the fruit significantly increased after gastric digestion, while these values decreased and then increased during intestinal digestion. Overall, the TPC in the intestinal digest was higher than that in the gastrointestinal digest, suggesting that gastrointestinal digestion increased the TPC in DJE. In addition, 30 phenolic compounds were identified during in vitro simulated gastrointestinal digestion. The antioxidant activity of the digested samples, as determined by the DPPH assay, varied with TPC, and there was a correlation between them, but that correlation was not as strong in ABTS. Therefore, this study suggests that in vitro digestion can facilitate the release of polyphenols in DJE with antioxidant effects. These findings provide important references for the potential benefits of polyphenols in jackfruit pulp for gastrointestinal health. Considering that polyphenols may undergo significant transformation during the process of digestion and absorption, and that the altered forms may exhibit distinct biological properties and effects, future research should also take into account their intestinal flora and metabolic behavior, which may influence health and disease treatment outcomes.

## Figures and Tables

**Table 1:** The analysis conditions of UPLC-ESI-QTOF-MS/MS.

Parameters	Agilent 1290 Infinity II UPLC, 6530B Hybrid Q-TOF-MS
Column	Agilent Zorbax Eclipse Plus C_18_ column (3.0 mm × 150 mm, 1.8 μm)
Mobile phase	0.1%formic acid in water (A) and acetonitrile (B)
Phase gradient	0–1.5 min, 5% B; 1.5–15 min, 5–60% B; 15–25 min, 60–100% B; 30–30.10 min, 100–5% B; 30.10–35 min, 5% B
Injection volume	3 μL
Column temperature	35 ℃
Flow rate	0.4 mL/min
Sheath gas	Helium
Sheath gas temperature	325 ℃
Sheath gas flow rate	11 L/min
Crash voltage	140 V

**Table 2:** UPLC-ESI-QTOF-MS characteristics of polyphenols and their metabolites in jackfruit pulp in vitro digestion.

Compd	Rt (min)	MS	MS/MS	Formula	Identification	Distribution					
						G0	G1	G2	I1	I2	I4
Hydroxybenzoic acids and derivatives											
1	3.172	171.0291	125	C_7_H_6_O_5_	Gallic acid			✓			
2	1.431	257.0657	153	C_9_H_10_O_5_	Syringic acid			✓			
Hydroxycinnamic acids and derivatives											
3	7.522	337.0906	147, 119	C_16_H_18_O_8_	5-p-Coumaroylquinic acid			✓			✓
4	7.323	399.0939	173, 179	C_16_H_18_O_9_	1-Caffeoylquinic acid			✓			
5	1.44	539.1212	163, 319	C_25_H_24_O_12_	(E,E)-3,5-di-O-Caffeoylquinic acid				✓		
6	1.674	367.1056	135	C_17_H_20_O_9_	3-O-Caffeoylquinic acid methyl ester				✓		
7	8.757	531.1475	204,163	C_26_H_26_O_12_	4,5-di-O-Caffeoylquinic acid ester				✓		✓
8	4.503	543.1557	326, 163	C_27_H_28_O_12_	Ethyl-3,4-dicaffeoylquinate					✓	
Flavanols											
9	1.563	353.0869	245	C_15_H_16_O_7_	(+)-Catechin hydrate			✓	✓		
10	8.304	441.0798	167, 137, 125	C_22_H_18_O_10_	(−)-Epicatechin gallate		✓				
11	10.161	459.0919	125, 137,139	C_22_H_18_O_11_	Gallocatechin			✓			
12	20.856	599.1153	539, 407, 285	C_30_H_24_O_12_	Procyanidin A1			✓	✓		
Flavonols											
13	1.58	397.0781	303, 301, 273	C_15_H_14_O_9_	Quercetin dihydrate						✓
14	2.284	789.2055	591, 489	C_33_H_40_O_22_	Quercetin-3-O-beta-D-glucose-7-O-beta-D-gentiobiosiden					✓	
15	1.567	447.4016	285	C_21_H_18_O_12_	Kaempferol-3-glucuronide			✓	✓		
16	20.865	595.1632	287, 285, 449	C_27_H_30_O_15_	Kaempferol 3-O-robinobioside						✓
17	15.968	625.1758	314	C_28_H_32_O_16_	Isorhamnetin-3-O-neohesperidoside	✓				✓	
18	16.206	553.0848	493	C_21_H_18_O_14_	Hibifolin	✓					
19	7.151	497.1851	438	C_25_H_26_O_7_	Papyriflavonol A			✓			
20	1.679	677.2371	351	C_33_H_40_O_15_	Sagittatoside A			✓			
Flavanones											
21	3.37	633.1822	494, 465, 431	C_28_H_34_O_15_	Hesperidin			✓	✓		
22	6.772	271.064	177,151, 107	C_15_H_12_O_5_	Naringenin			✓			✓
23	7.3	373.1289	181, 161	C_18_H_18_O_5_	Naringenin trimethyl ether			✓			
24	5.147	331.0828	193, 93	C_16_H_14_O_5_	5-O-Methylnaringenin					✓	
25	29.446	467.1715	367, 45, 123	C_25_H_26_O_6_	Kuwanol C			✓			
Flavones											
26	15.971	615.1728	593	C_28_H_32_O_14_	Fortunellin		✓				
27	7.301	395.1096	342, 357	C_20_H_20_O_7_	Tangeretin				✓		
28	6.347	553.1154	521, 477	C_31_H_20_O_10_	Bilobetin	✓		✓			✓
29	2.374	289.0703	271, 243, 153	C_15_H_12_O_6_	Aromadendrin				✓		
30	2.469	463.0875	162	C_21_H_20_O_12_	6-Hydroxyluteolin-7-glucoside						✓

G0: Gastric digestion 0 h, G1: Gastric digestion 1 h, G2: Gastric digestion 2 h, I1: Intestinal digestion 1 h, I2: Intestinal digestion 2 h, I4: Intestinal digestion 4.

## Data Availability

Data is contained within the article.

## Supplementary Materials

The following supporting information can be downloaded at: https://www.mdpi.com/article/10.3390/antiox13010037/s1, Table S1. Preparation of electrolyte solutions and simulated digestive fluids. Figure S1. Positive and negative total ion chromatogram of DJE. Figure S2. MS spectra and structural formulae of 30 compounds identified in JPE after in vitro simulated gastrointestinal digestion.
