# Supplementary material for: Changes in Phenolic Compounds and Antioxidant Capacity of Artocarpus heterophyllus Lam. (Jackfruit) Pulp during In Vitro Gastrointestinal Digestion
Source: Antioxidants (Basel). 2023 Dec 23;13(1):37. doi: 10.3390/antiox13010037 (PMC10812572; doi:10.3390/antiox13010037)
Supplement: Supplementary file 1 [file antioxidants-13-00037-s001.zip › antioxidants-2734457-supplementary.pdf]

## Supplementary Material

**Table S1.** Preparation of electrolyte solutions and simulated digestive fluids

| | | | SSF[1] (pH 7) | | SGF[2] (pH 7) | | SIF[3] (pH 7) | |
|---|---|---|---|---|---|---|---|---|
| | Stock concentrations | | mL of Stock added to prepare 0.4 L (1.25 x) | Final salt conc. in SSF | mL of Stock added to prepare 0.4 L (1.25 x) | Final salt conc. in SGF | mL of Stock added to prepare 0.4 L (1.25 x) | Final salt conc. in SIF |
| Salts | g/L | mol/L | mL | mmol/L | mL | mmol/L | mL | mmol/L |
| KCl | 37.3 | 0.5 | 15.1 | 15.1 | 6.9 | 6.9 | 6.8 | 6.8 |
| $KH_2PO_4$ | 68.0 | 0.5 | 3.7 | 3.7 | 0.9 | 0.9 | 0.8 | 0.8 |
| $NaHCO_3$** | 84.0 | 1.0 | 6.8 | 13.6 | 12.5 | 25.0 | 42.5 | 85.0 |
| NaCl | 117.0 | 2.0 | - | - | 11.8 | 47.2 | 9.6 | 38.4 |
| $MgCl_2(H_2O)_6$ | 30.5 | 0.15 | 0.5 | 0.15 | 0.4 | 0.12 | 1.1 | 0.33 |
| $(NH_4)_2CO_3$ | 48.0 | 0.5 | 0.06 | 0.06 | 0.5 | 0.5 | - | - |
| $CaCl_2(H_2O)_2$ | 44.1 | 0.3 | - | 1.5 | - | 0.15 | - | 0.6 |
| $H_2O_{up}$ | - | - | 373.84 | - | 367.0 | - | 339.2 | - |

* [1] Simulated salivary fluid; [2] Simulated gastric fluid; [3] Simulated intestinal fluid.

** $CaCl_2(H_2O)_2$ was added before digestion to prevent the formation of precipitation of calcium ions.

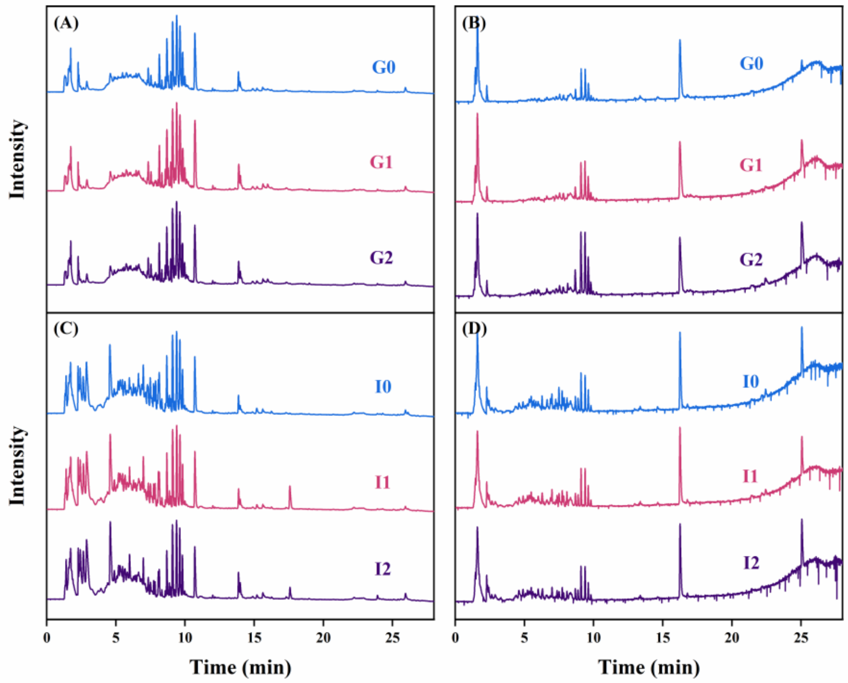


**Figure S1.** Positive (A, C) and negative (B, D) total ion chromatogram of DJE. G0: Gastric digestion 0 h, G1: Gastric digestion 1 h, G2: Gastric digestion 2 h, I1: Intestinal digestion 1 h, I2: Intestinal digestion 2 h, I4: Intestinal digestion 4 h.

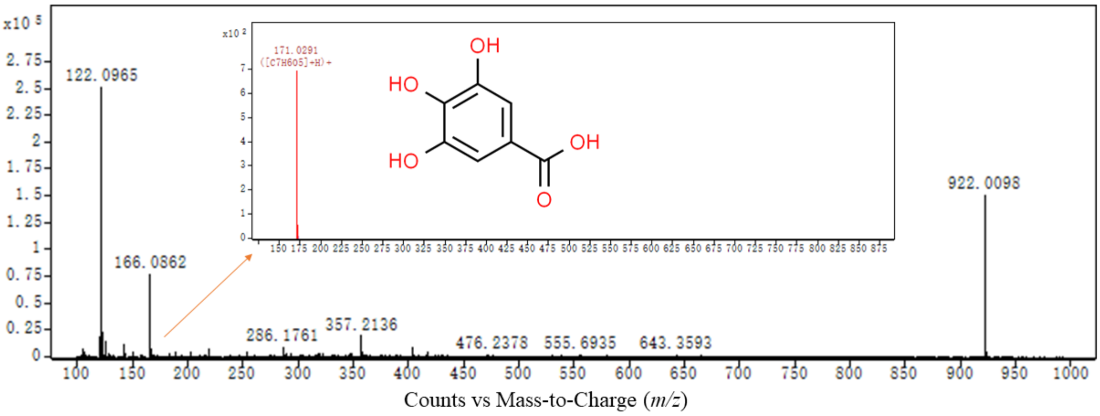


(compound 1)

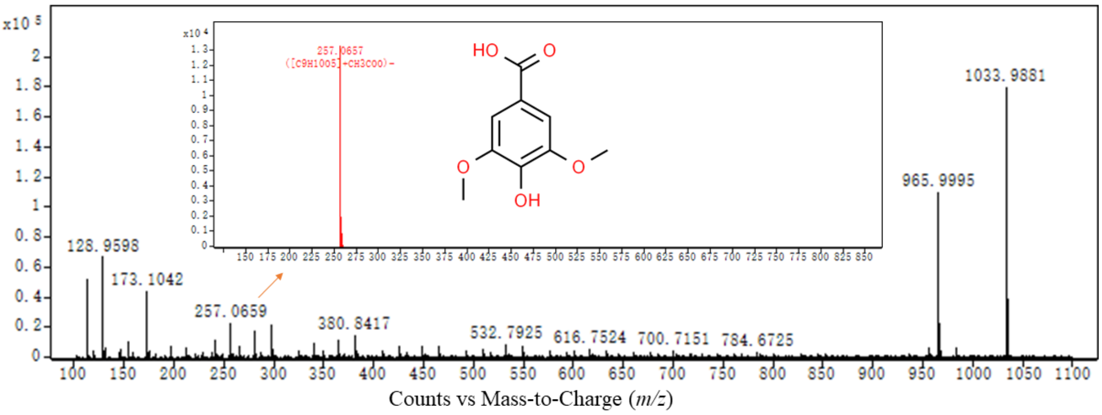


(compound 2)

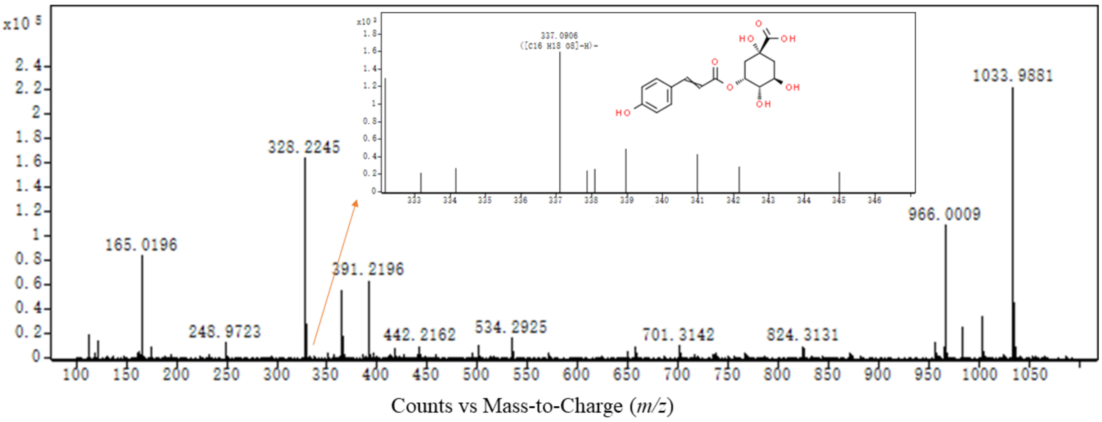


(compound 3)

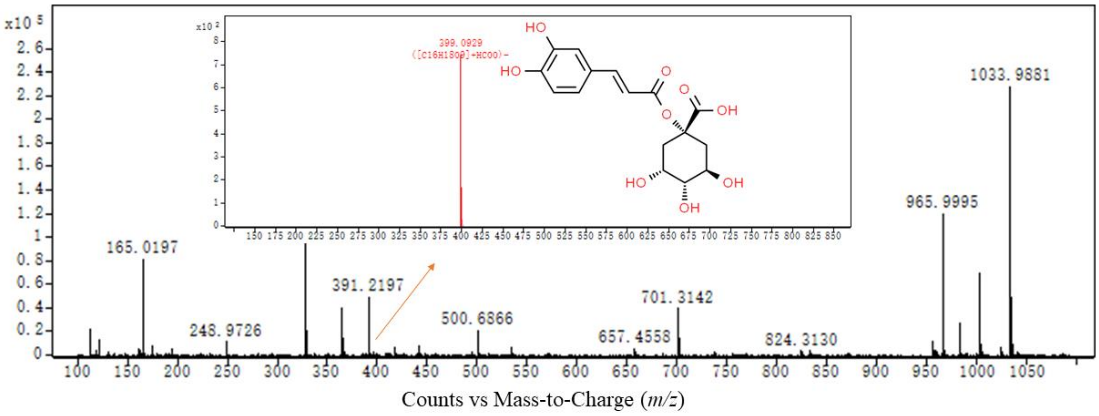

(compound 4)

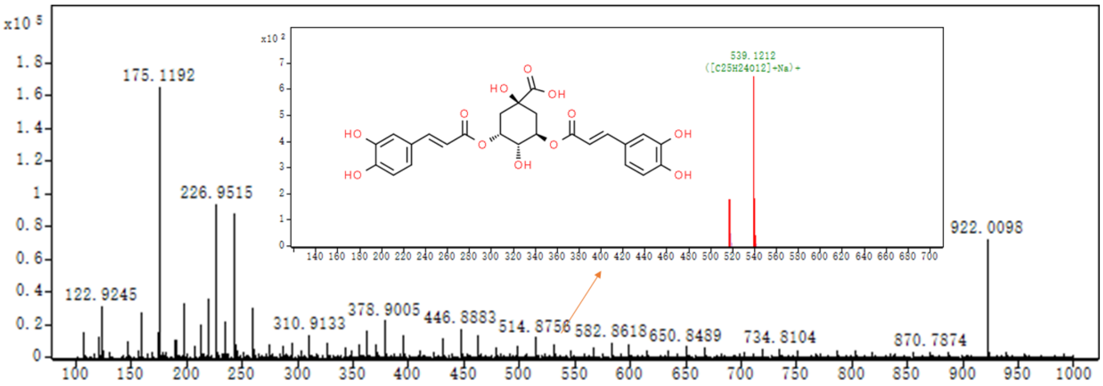


Counts vs Mass-to-Charge (*m/z*)

(compound 5)

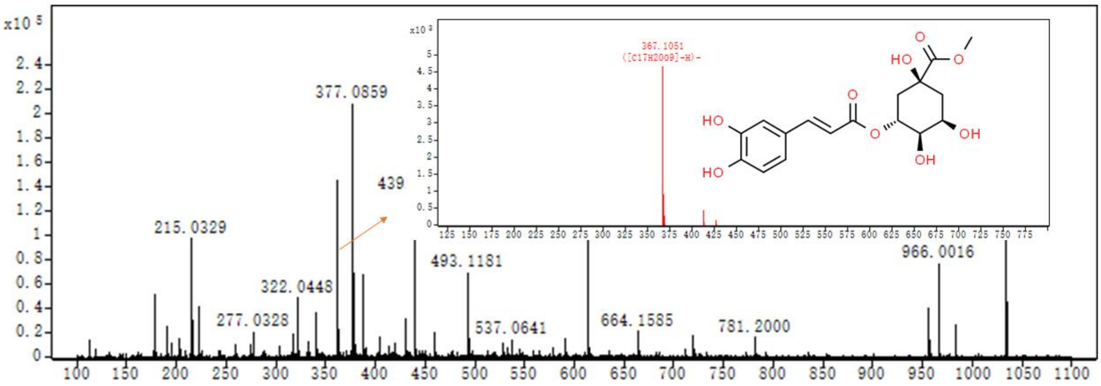


Counts vs Mass-to-Charge (*m/z*)

(compound 6)

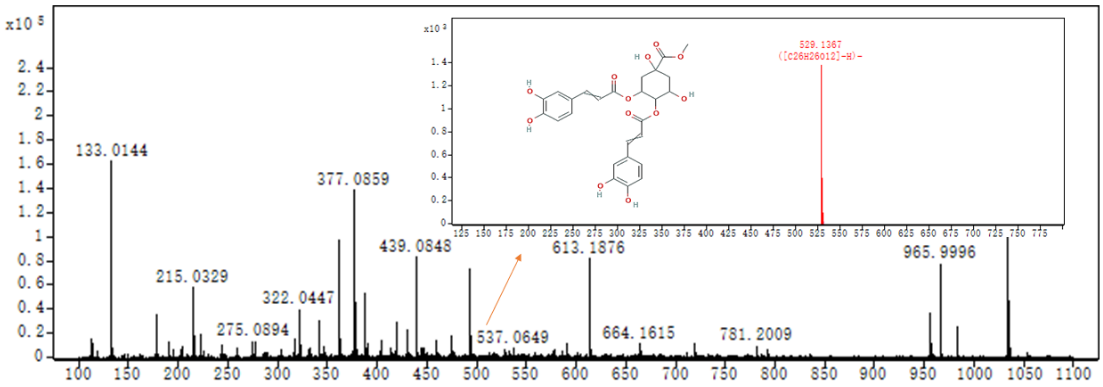


Counts vs Mass-to-Charge (*m/z*)

(compound 7)

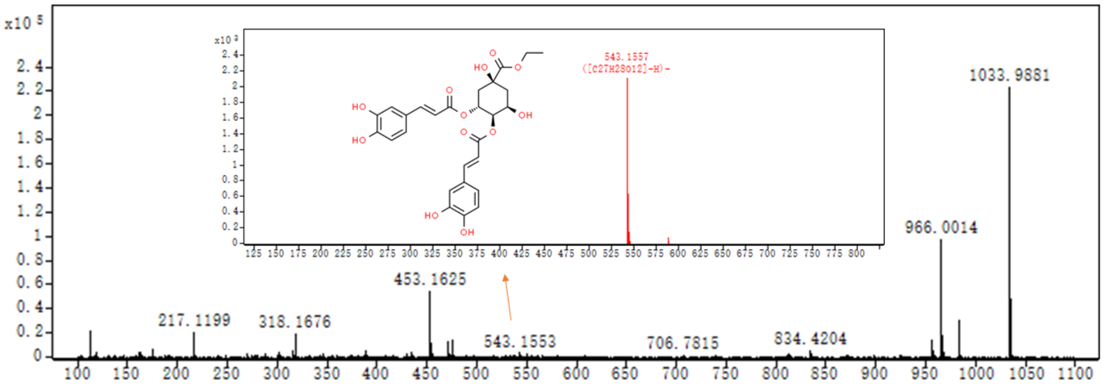


Counts vs Mass-to-Charge (*m/z*)

(compound 8)

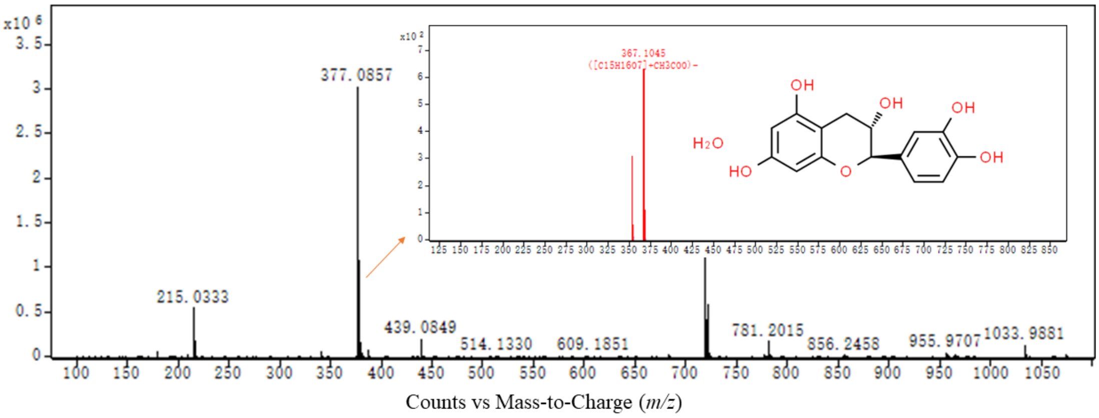


(compound 9)

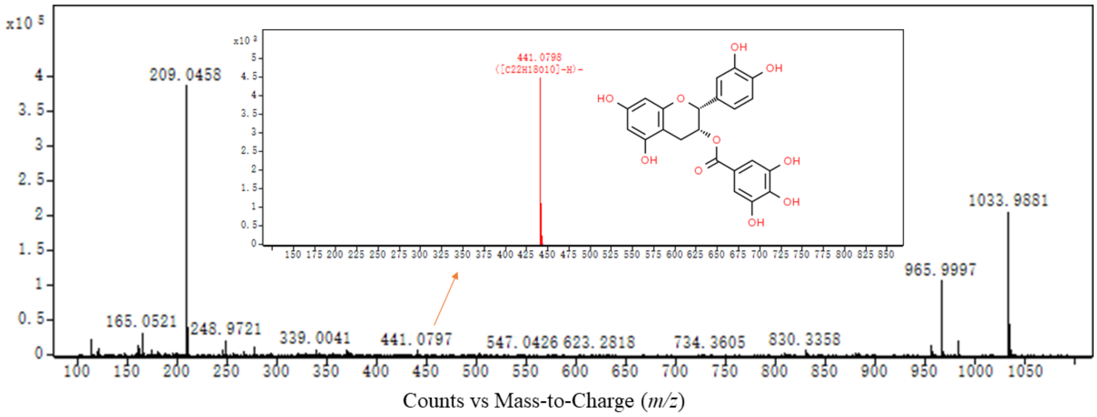


(compound 10)

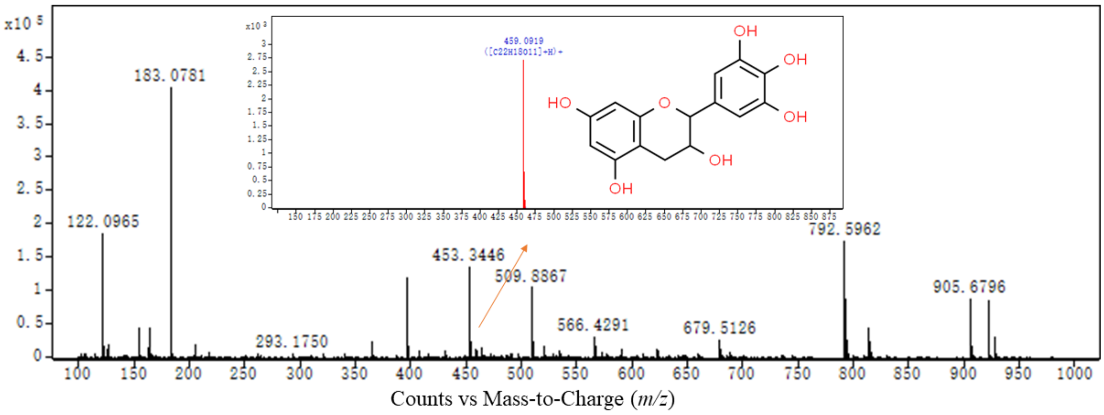


(compound 11)

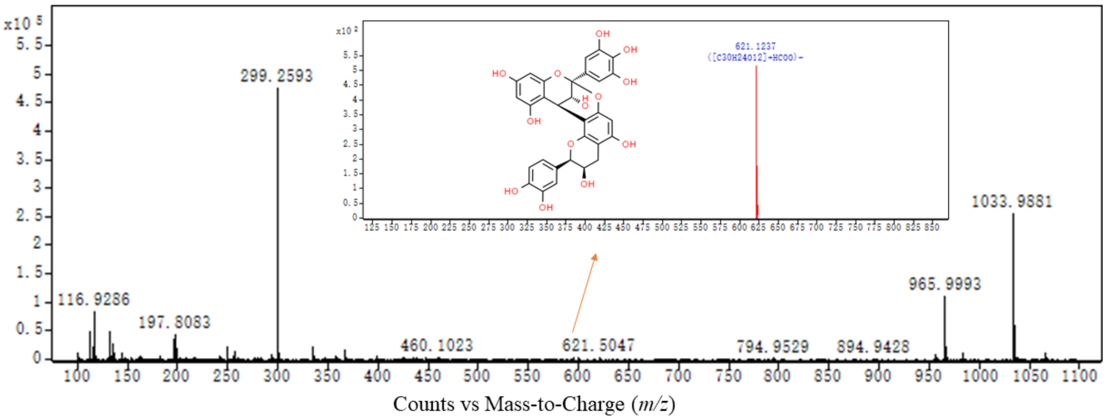

(compound 12)

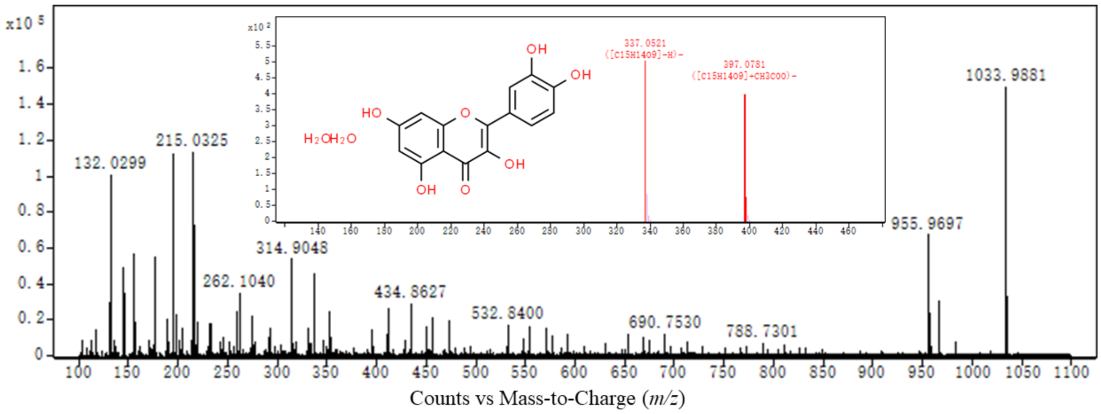


(compound 13)

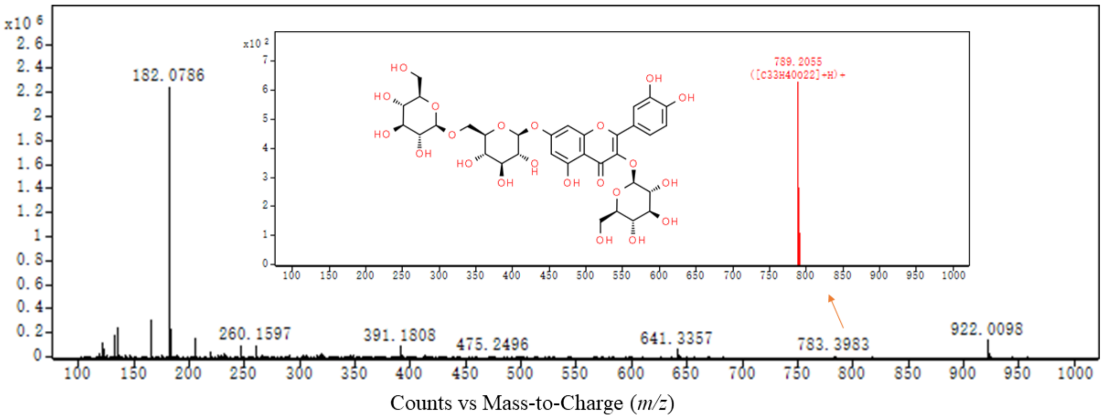


(compound 14)

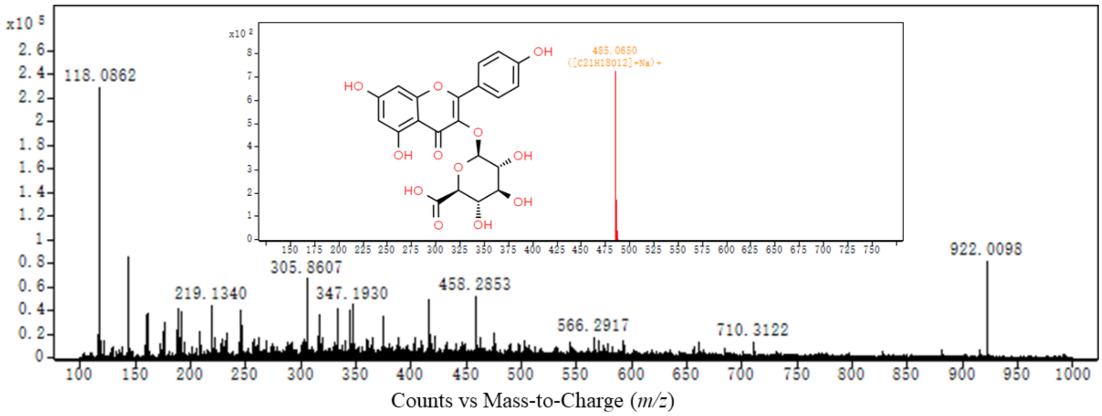


(compound 15)

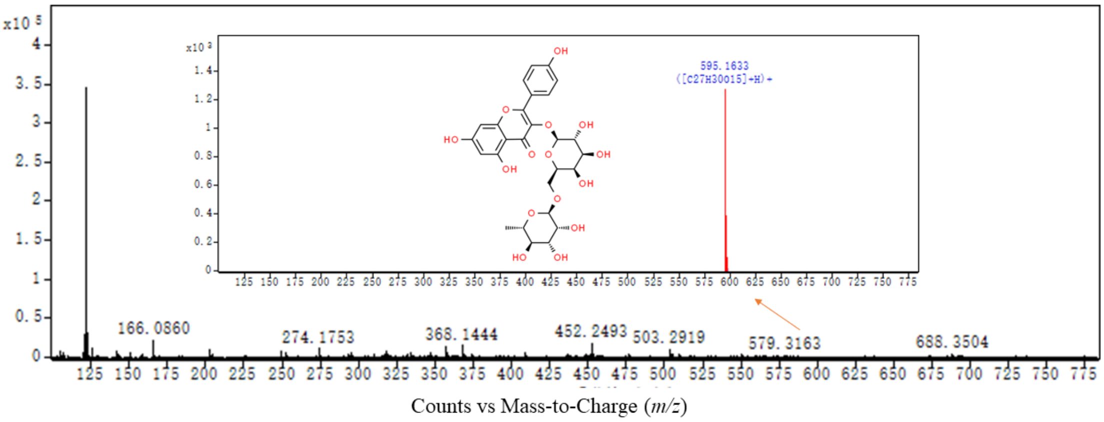

(compound 16)

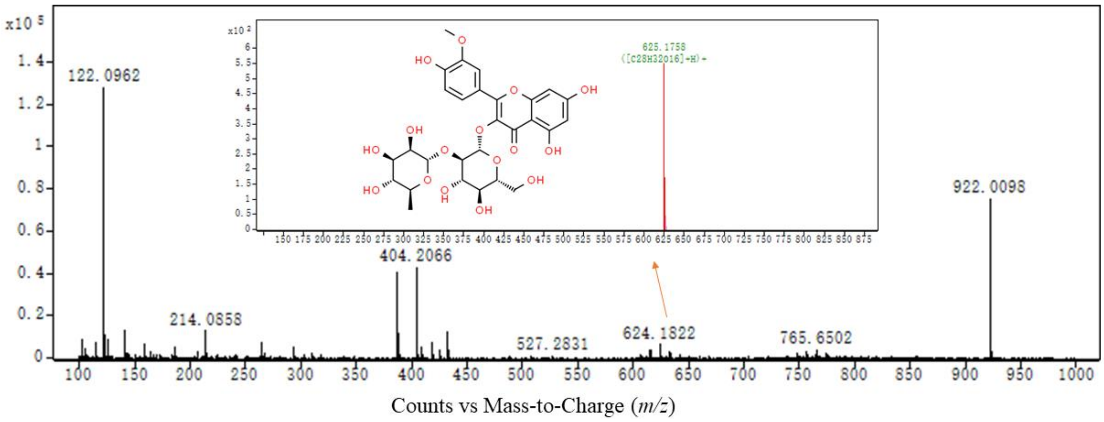


(compound 17)

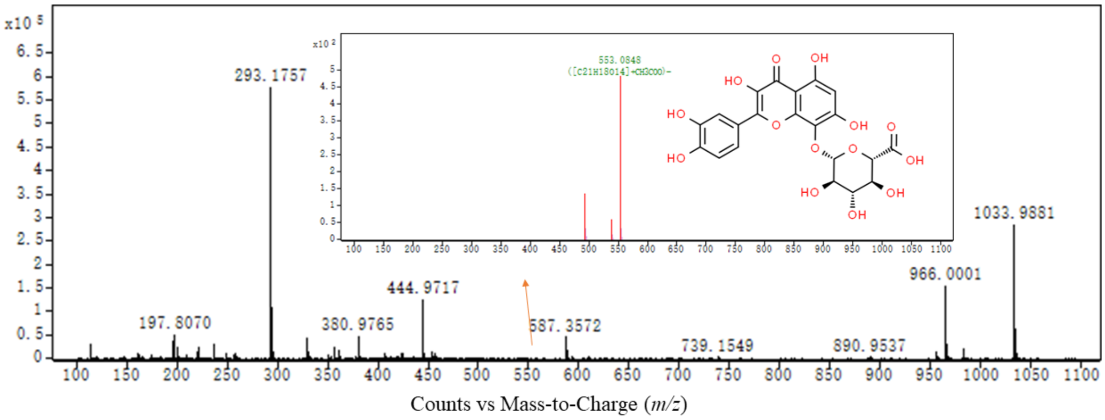


(compound 18)

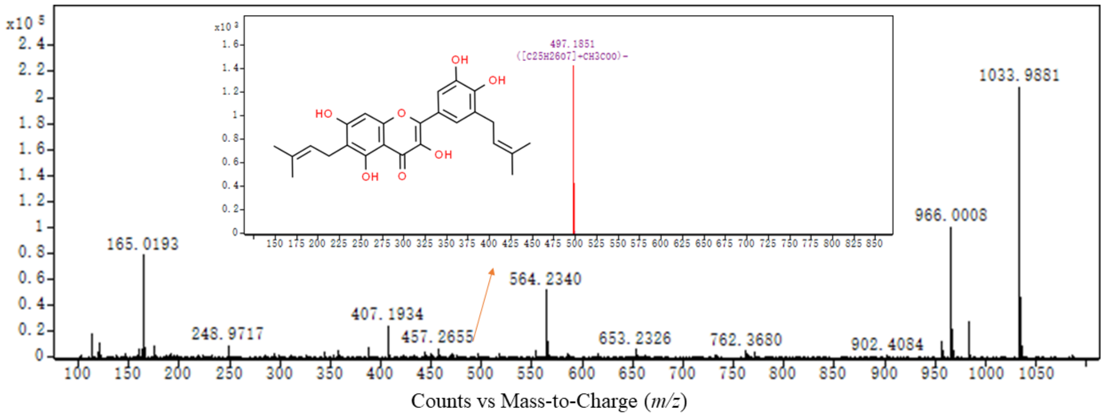


(compound 19)

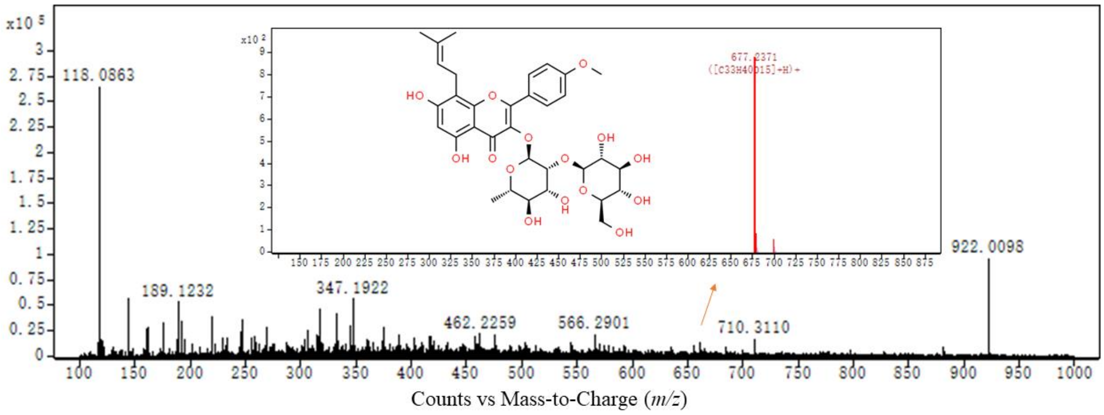

(compound 20)

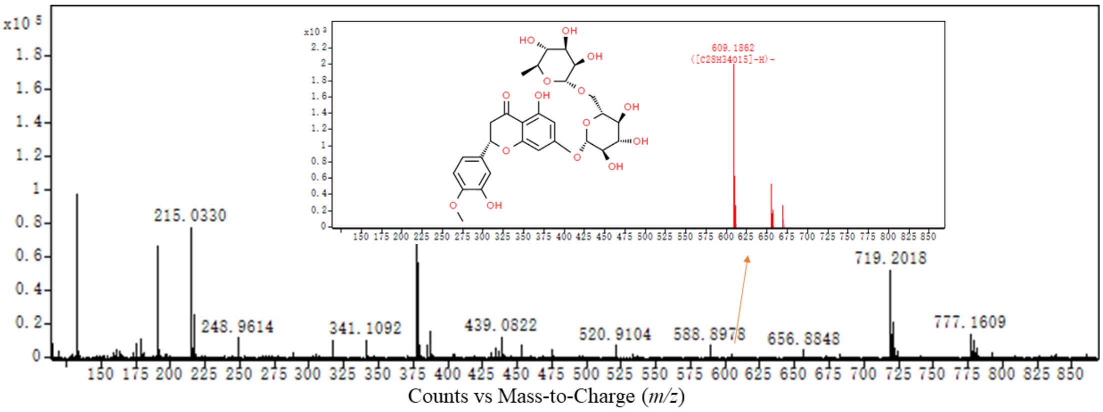


(compound 21)

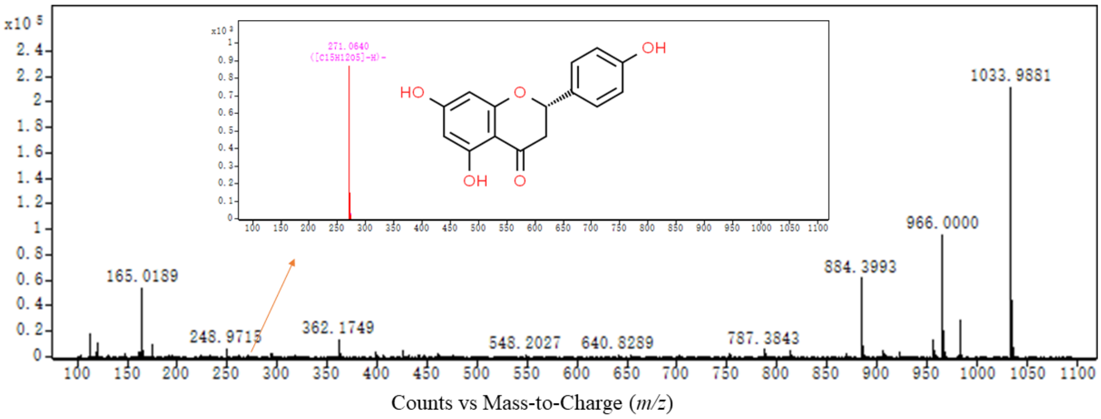


(compound 22)

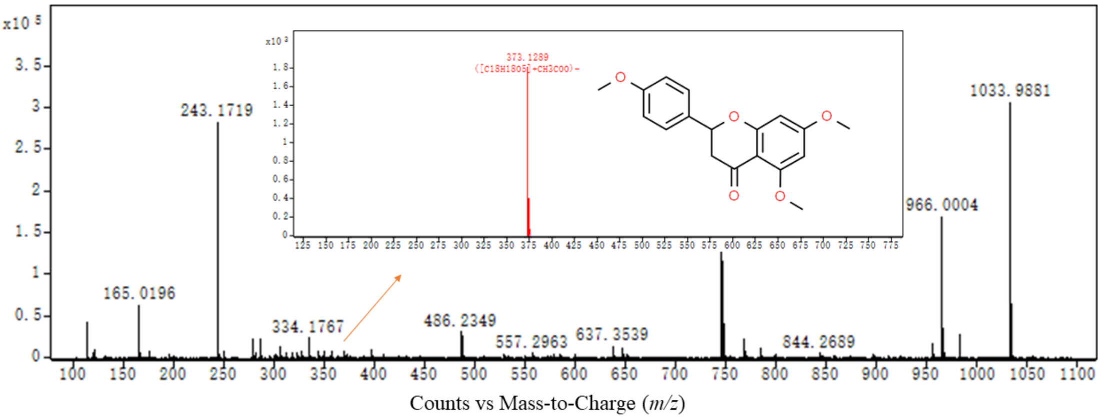


(compound 23)

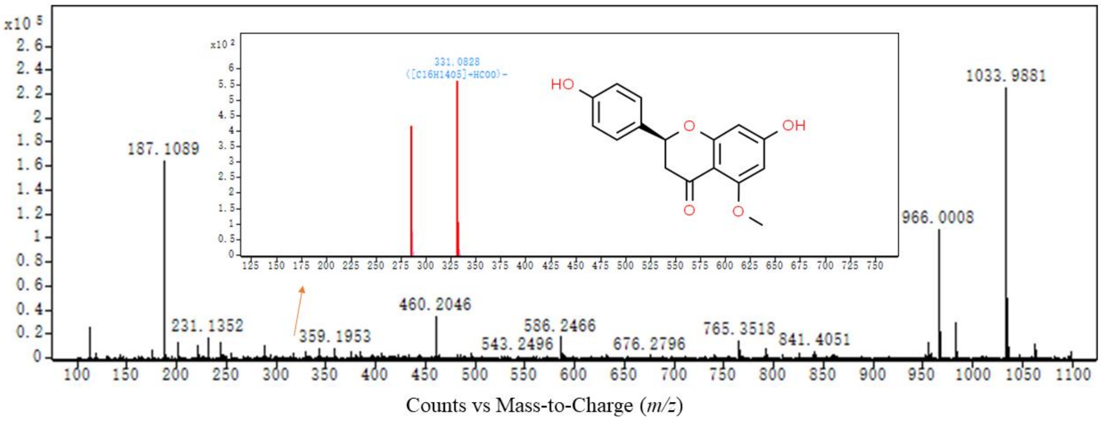

(compound 24)

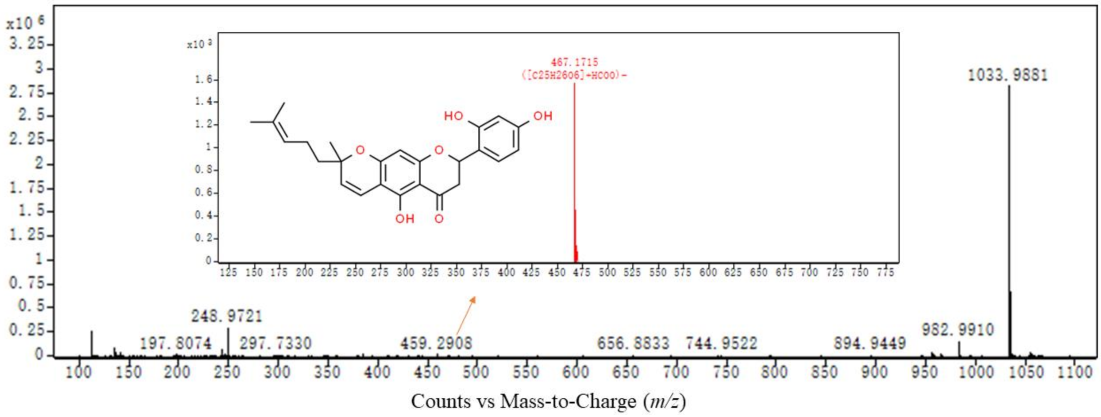


(compound 25)

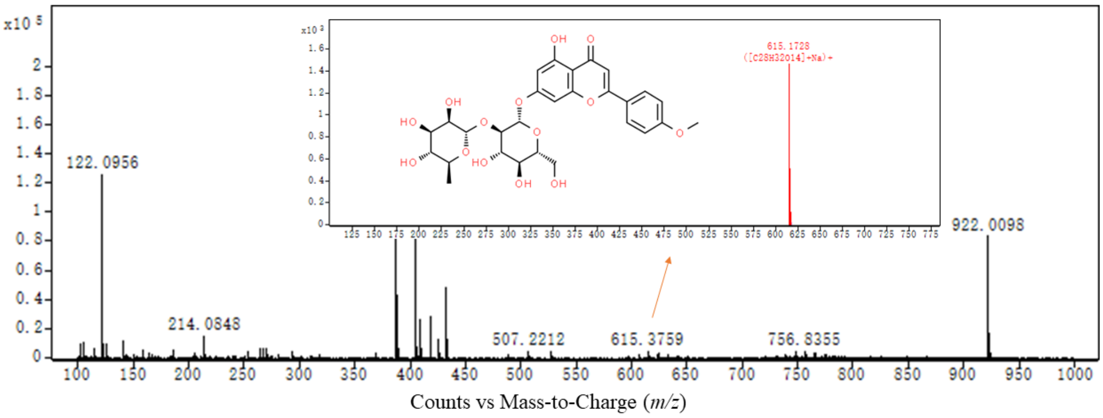


(compound 26)

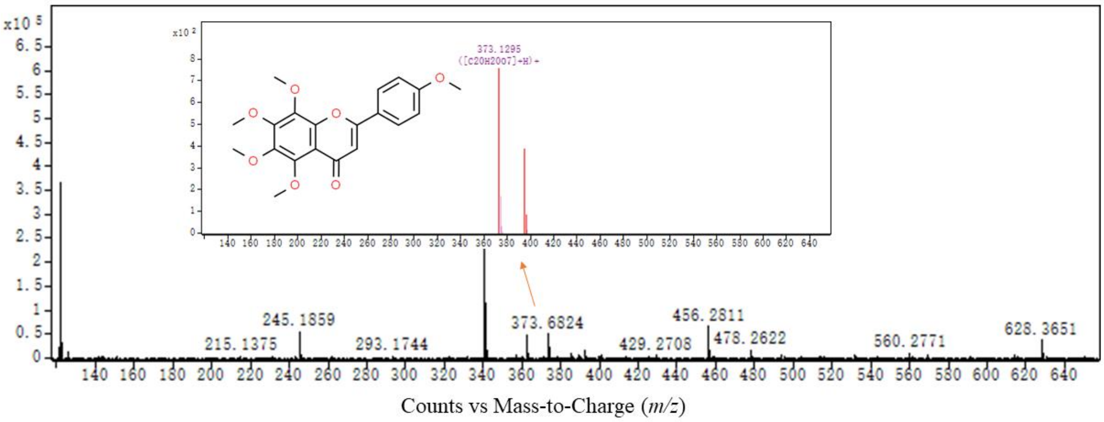


( compound 27)

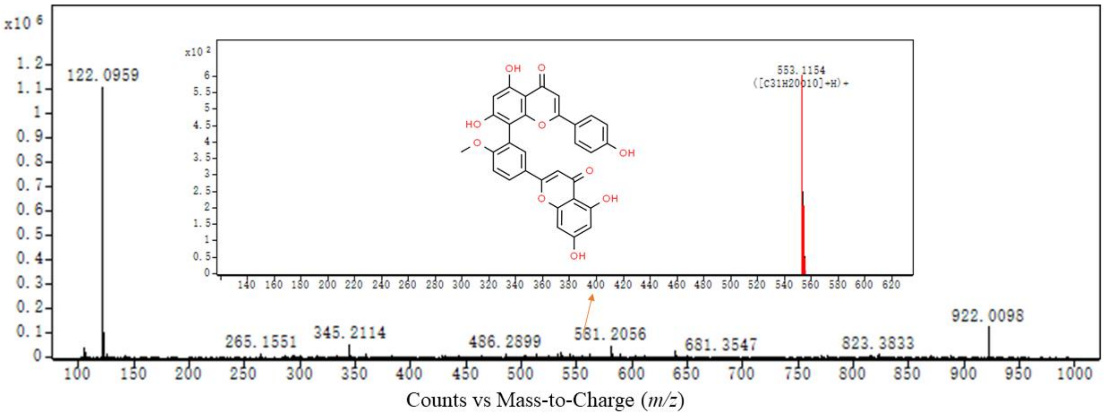

(compound 28)

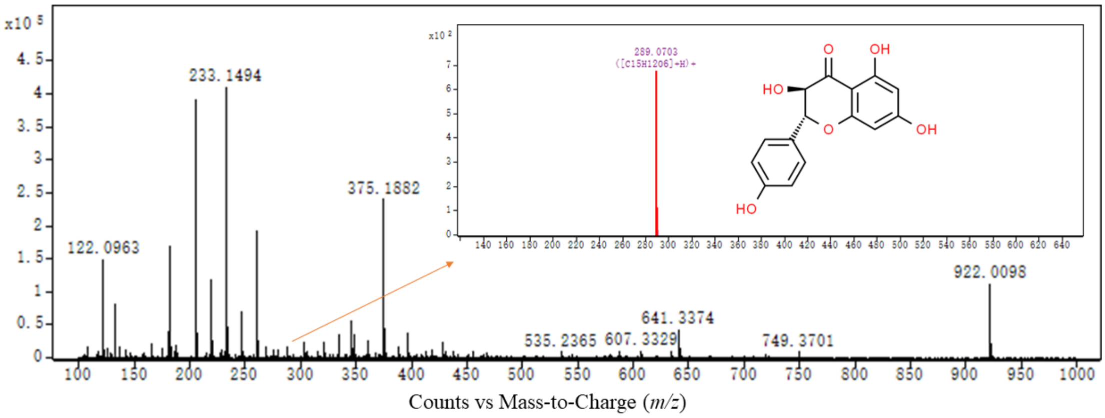


(compound 29)

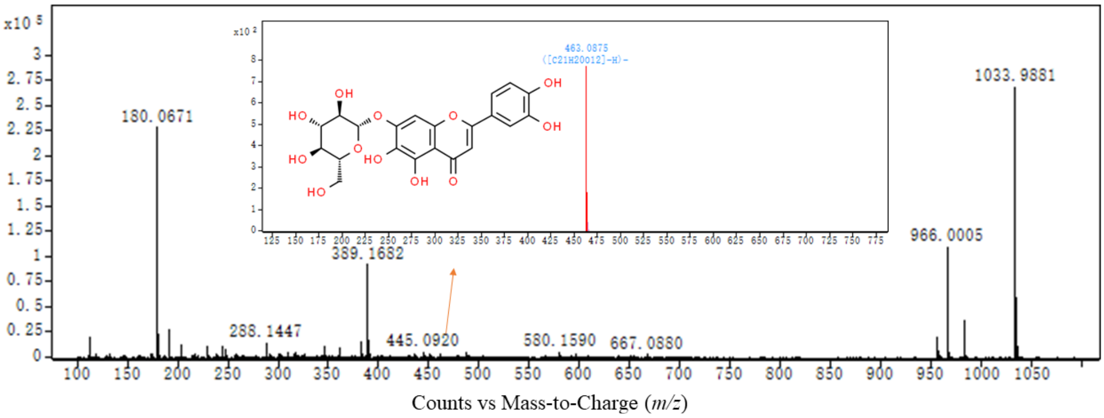


(compound 30)

**Figure S2.** MS spectra and structural formulae of 30 compounds identified in JPE after *in vitro* simulated gastrointestinal digestion
